# No Association between Low-Calorie Sweetener (LCS) Use and Overall Cancer Risk in the Nationally Representative Database in the US: Analyses of NHANES 1988–2018 Data and 2019 Public-Use Linked Mortality Files

**DOI:** 10.3390/nu14234957

**Published:** 2022-11-22

**Authors:** Victor L. Fulgoni, Adam Drewnowski

**Affiliations:** 1 Nutrition Impact LLC, Battle Creek, MI 49014, USA; 2 Center for Public Health Nutrition, University of Washington, Seattle, WA 98195, USA

**Keywords:** low-calorie sweeteners (LCS), NHANES 1988–2018, mortality files, socioeconomic status, smoking, HEI-2015, cancer mortality, aspartame, saccharin

## Abstract

Low-calorie sweeteners (LCS) serve to replace added sugars in beverages and foods. The present goal was to explore any potential links between LCS use and cancer risk using the nationally representative National Health and Nutrition Examination Surveys 1988–2018 linked to 2019 Public-Use Linked Mortality Files. Analyses were based on dietary intakes from 1988–1994 NHANES (*n* = 15,948) and 1999–2018 NHANES (*n* = 48,754) linked to mortality data. The 1988–1994 NHANES separated aspartame from saccharin consumption; later data did not. LCS consumers were more likely to be older, female, non-Hispanic White, and with higher education and incomes compared to nonconsumers. LCS consumers were less likely to smoke and had higher HEI-2015 scores indicating higher-quality diets. In the cross-sectional NHANES data, LCS use was associated with higher BMI and higher prevalence of obesity and diabetes. There was no indication that aspartame, saccharin, or all LCS had any impact on overall cancer mortality. By using nonconsumers as the reference group, the hazard ratio (95th confidence interval, CI) group trend for tertiles of LCS use for 1988–1994 for aspartame was 1.00 (0.89–1.12), for saccharin 0.96 (0.79–1.10), and for 1988–2018 for all LCS was 0.92 (0.88–1.101). The null group trend effects were seen for analyses stratified by age/gender. The present analyses confirm past US-based reports that LCS use was associated with higher socioeconomic status, lower prevalence of smoking, and generally higher-quality diets. No association with cancer mortality was observed.

## 1. Introduction

By replacing added sugars in beverages and some foods, low-calorie sweeteners (LCS) reduce sugar calories, maintain palatability, and may assist in the management of body weight [1,2]. Based on analyses of nationally representative National Health and Nutrition Survey (NHANES) cycles for 2009–2012, LCS were consumed by 41.4% of US adults [3]. LCS consumption was higher among individuals with obesity as compared to overweight and normal-weight individuals and was higher among women than among men [3]. The frequency of LCS consumption increased with body weight [3]. The nutritional benefits and risks related to LCS use have been addressed in numerous reviews [4,5,6].

The association between LCS use and higher body weight, normally seen in cross-sectional studies, has at times been taken to suggest that LCS use may lead to obesity, type 2 diabetes, and the metabolic syndrome [7,8,9,10]. Among proposed metabolic mechanisms were heightened response to sweet taste [11], impaired satiety and metabolic derangements [12], and altered gut microbiota [13]. Despite having been disproved multiple times, many such hypotheses still persist [14,15].

The issue of reverse causality has also been addressed. The NHANES database includes a 10-year weight history questionnaire along with retrospective data on the intent to lose or maintain weight during the preceding 12 months [16]. Analyses of behavioral data showed that those adults who had tried to lose weight during the previous 12 months were more likely to consume LCS in the form of beverages, tabletop LCS, and LCS foods [16]. Current LCS use was further associated with a past 10-year history of weight fluctuations, including both weight loss and weight regain [16]. It would appear that concern with body weight leads to LCS use rather than the other way around.

Fewer human studies have explored any potential association between LCS use and cancer risk. In 2006, the National Cancer Institute concluded that increasing consumption of aspartame-containing beverages was not associated with the development of lymphoma, leukemia, or brain cancer [17]. A 2013 review of epidemiologic evidence also found no association between aspartame use and cancer risk [18]. The European Food Safety Authority (EFSA) [19], and the French Agence Nationale de Securite’ Sanitaire de l’Alimentation, de l’Environnement et du Travail (ANSES) [20] have not found a link between aspartame consumption and cancer at the current levels of exposure. The calls to re-evaluate LCS safety in cancer development are based on in vitro studies and on data from mice and rats, much of which has been evaluated previously by public health and regulatory agencies [21], and on very limited human data [22].

A recent paper, based on the NutriNet Santé cohort in France, reported that major consumers of LCS had higher risk of overall cancer [23]. Separating self-reported LCS use by brand, that study reported higher risks for overall cancer for aspartame and acesulfame K and higher risk for obesity-related cancers for aspartame only [23]. However, despite its large size, the largely female NutriNet Santé cohort appears to be a non-representative and potentially highly biased sample of convenience [24].

The present study used multiple cycles of the nationally representative NHANES data for the US for the period 1988–2018 [25] to explore the association between LCS use and overall cancer risk. Dietary intakes from the first 24 h dietary recall were merged with measured body weights, health history data and with 2019 public-use linked mortality files [26]. We hypothesized that LCS use would be associated with higher body weights and with certain obesity-related health conditions but that there would be no association of LCS use with cancer-related mortality.

## 2. Materials and Methods

### 2.1. Data Source and Population

Data analyses were based on multiple cycles of the nationally representative cross-sectional National Health and Nutrition Examination Survey (NHANES) for years 1988–1994 [27] and 1999–2018 [25]. The NHANES is the main source of dietary surveillance data in the US and serves to inform the Dietary Guidelines for Americans and other federal and state food and nutrition policies [25]. The dietary recall component currently uses a multipass method to measure all foods consumed midnight-to-midnight during the day prior to data collection [28]. The present analyses were based on 15,948 participants aged >19 years in 1988–1994 NHANES (then called NHANES III) [23] and on 47,854 participants in 1999–2018 NHANES [25] who completed a valid 24-h recall, as defined by National Center for Health Statistics staff. The necessary IRB approval for NHANES had been obtained by the National Center for Health Statistics (NCHS) [29]. Adult participants provided written informed consent. All NHANES data are publicly available [25].

### 2.2. The Food and Nutrient Database for Dietary Studies (FNDDS)

The Food and Nutrient Database for Dietary Studies (FNDDS) maintained by the US Department of Agriculture is used to calculate energy and nutrient content of foods consumed by NHANES participants [30]. The foods are aggregated into multiple food groups, subgroups, and categories by using What We Eat in America coding schemes [30]. The FNDDS does not automatically code beverages and foods as containing LCS, and a custom coding approach is required. All food items in the individual foods file needed to be examined and queried based on the food description, energy density (kcal/100 g), and total and added sugars content in g per 100 g and per average consumption report. Food categories of interest were diet beverages, including diet sodas and diet beverages (fruit based and other), tabletop sweeteners, and low-calorie sugar free foods such as yogurts.

The most common LCS beverages were soft drinks, described as cola-type or fruit-flavored drinks that were further described as sugar-free, low-calorie, or diet [31]. Also included were teas pre-sweetened with LCS. Making a much smaller contribution to LCS use were diet yogurt, ice cream, grain-based desserts, and candies [31]. The weight of LCS beverages and foods was calculated as the weight in grams of the entire diet beverage or diet food. Tabletop LCS were a special category of sugar substitutes (WWEIA category 8804). Tabletop LCS were coded as powder products used to sweeten beverages, coffee, or tea. For these powders, the weight of the beverage to which the LCS were added was set at 170 g (grams of 6 oz coffee) plus the negligible weight of the powder itself. Less than 2% (*n* = 165) of food items were classified as containing LCS (see Supplemental Table S1 for full list). The FNDDS does not separate LCS by brand name (e.g., aspartame, sucralose, saccharin). Only in NHANES 1988–1994, aspartame and saccharin intakes were measured directly in mg/d.

LCS consumers were assigned to three categories based on consumption tertiles. Those were based on tertiles of aspartame and saccharin consumption in the NHANES 1988–1994 analyses and on tertiles of gram weight of LCS beverages and foods in NHANES cycles 1988–1994, 1999–2018, and 1988–2018

### 2.3. Health Behaviors and Diet Quality Measures

Data on smoking status and physical activity were obtained by self-report. Smoking status was categorized into: Current, former, or never smokers. Self-reported physical activity was categorized into sedentary, moderate, and vigorous. Dietary intake data from 24 h dietary recalls were used to construct Healthy Eating Index (HEI 2015) scores. The HEI 2015 [32], developed by the U.S. Department of Agriculture, is a measure of diet quality as determined by compliance with the Dietary Guidelines for Americans [33]. The HEI 2015 is a 12-component, 100-point scale wherein higher scores are associated with better adherence to dietary guidelines [33]. Alcohol use was assessed through 24-h recall.

### 2.4. Comparing LCS Consumers and Nonconsumers by Demographics and Diet Quality Measures

LCS consumers and nonconsumers were compared for NHANES cycles 1988–1994, and 1999–2018 on a number of demographic and dietary variables by using regression analyses adjusting for the complex sampling plan of NHANES (i.e., using primary sampling units and strata) and exam weights for NHANE 1988–1994 and Day 1 dietary weights for 1999–2018.

### 2.5. Analytical Methods to Assess Cancer Mortality Risk

Excluded from mortality analyses were NHANES participants who reported having certain chronic diseases, and those with missing covariate data needed for analysis. The exclusion numbers are shown in Table 1. For the 19+ age group, cancer mortality was 6.97 ± 0.36% for the 1988–1994 sample, 1.80 ± 0.08% for 1999–2018, and 2.85 ± 0.10% for the 1988–2018 sample. For the 51+ years groups, comparable mortality percentages were 15.84 ± 0.82% for the 1988–1994 sample, 4.33 ± 0.23% for 1999–2018, and 6.31 ± 0.26% for the 1988–2018 sample. The average years of follow-up for those aged 19+ years were 25.1 for 10.3 for 1988–1994, and 13.3 for 1999–2018. Comparable years of follow-up for the 51+ years age group were 11.1, 19.3, and 9.4 years, respectively.

Cox proportional hazards regression was used to estimate the hazard ratios (HRs) and 95th percentile lower and upper confidence levels for cancer mortality. HRs were estimated by assigning nonconsumers of aspartame/saccharin/LCS to the reference group (HR = 1.0). LCS consumers were then split by tertiles of consumption T1, T2, and T3. For analytical purposes, dietary intakes data were stratified by gender (male, female) age group (19–50; >50 years); race/ethnicity (non-Hispanic White; non-Hispanic Black, Mexican American, other Hispanic, and other); education (high school or less; some college; and college graduate, strata corresponding to <12 years, 12–16 years, and >16 years of education), and by poverty-to-income-ratio or PIR (cut points: <1.35, 1.35 to 1.85, and >1.85).

Multivariable adjusted HRs were determined by using SAS 9.4 PROC SURVEYPHREG with age, sex, race/ethnicity, education, current smoking status (Y/N), alcohol consumption, physical activity level, and BMI (continuous) as covariates. Analyses were presented separately by sex and stratified by age group (19+ years, 19–50 years, and 51+ years). For all analyses, *p* < 0.05 was considered statistically significant.

All analyses were adjusted for the complex sample design of NHANES with NHANES 1988–1998 exam weights and NHANES 1999–2018 dietary weights. When data for NHANES 1988–1994 data and NHANES 1999–2018 were combined, the sample weights for the combined dataset were constructed by treating NHANES 1988–1994 data as similar to three NHANES two-year cycles as for years 1999–2018. The NHANES 1988–1994 database for a six-year period had a number of sample observations that was similar to three NHANES cycles for 1999–2018. When datasets were combined, then the weights for the subjects from NHANES 1988–1994 were 3/13 times the weight given in NHANES 1988–1998 and the weights for subjects from NHANES 1999–2018 were 1/13 times the weight given in NHANES 1999–2018. Per NHANES analytical guidelines, when NHANES 1999–2000 and NHANES 2001–2002 were included in the analysis, the four-year weights as given by NHANES were used.

## 3. Results

### 3.1. Participant Characteristics for 1988–1994 and 1999–2018 NHANES Surveys

Participant characteristics are shown in Table 2. The NHANES sample for 1988–2018 was composed of male and female adults, aged >19 years, mean age 46.5 years, with about 70% identifying as non-Hispanic White. Two-thirds of the sample had household PIR > 1.85. College graduates were over 25% of the sample. The prevalence of current smoking was about 22%; obesity percentage was about 330%, and mean BMI was 28.3.

### 3.2. Characteristics of LCS Consumers and Non Consumers in 1988–1994 and 1999–2018 NHANES

LCS consumers and nonconsumers are compared in Table 3. In both NHANES series, LCS consumers were more likely to be female, non-Hispanic White, with higher incomes (PIR > 1.85) and with college education. Importantly, LCS consumers were less likely to be current smokers (15.51% vs. 23.98% for nonconsumers). As expected, LCS use was associated with higher BMI (29.87 vs. 27.86 for nonconsumers); with higher prevalence of obesity (41.95% vs. 30.02% for nonconsumers) and with much higher self-reported prevalence of diabetes (19.18% vs. 7.23% for nonconsumers in the 1999–2018 NHANES).

### 3.3. Comparing LCS Consumers and Nonconsumers by Diet Quality (HEI-2015)

**Figure 1:**
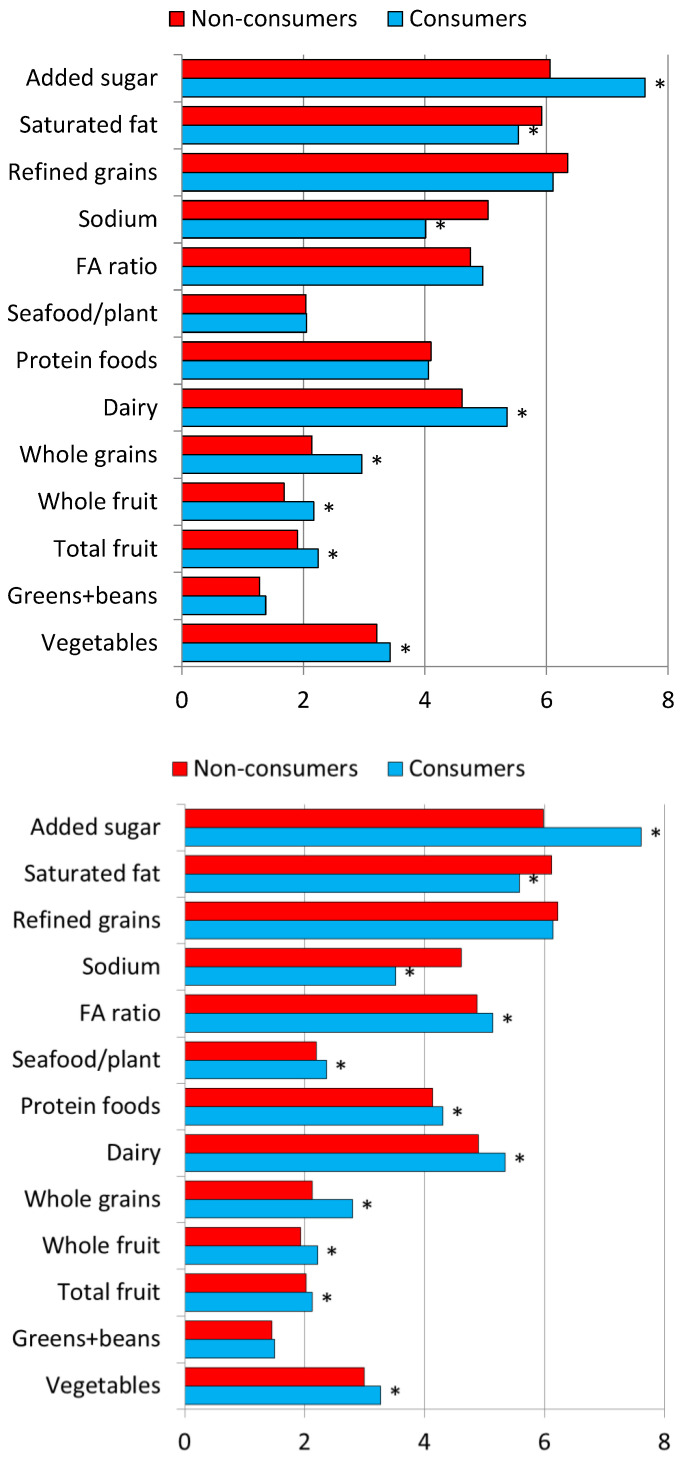
Comparing consumers and nonconsumers of LCS by HEI-2015 diet quality scores in 1988–1994 NHANES (top) and 1999–2018 NHANES (bottom). * Significantly different from nonconsumers, *p* < 0.05.

LCS consumers had significantly higher HEI 2015 scores than did nonconsumers. This effect was significant for both 1988–1994 and 1999–2018 cycles. Figure 1 shows that LCS consumers had significantly higher HEI subscores for added sugars but also for total vegetables, greens and beans, total fruit (*p* < 0.004), whole fruit (*p* < 0.001), whole grains, and dairy subscores compared to nonconsumers. On the other hand, LCS consumers had lower (i.e., less favorable) HEI subscores on saturated fat and sodium compared to nonconsumers. Comparable effects were obtained for the 1988–1994 and 1999–2018 NHANES cycles, as shown in Figure 1. 

Figure 1 also shows that the association between LCS consumption and higher HEI-2015 scores was largely driven by a more favorable subscore (meaning lower consumption) for added sugars. Once added sugars scores were removed from the analysis, the differences in total HEI-2015 scores were still significant. but the difference in total scores was now smaller. For 1988–1994 NHANES, HEI-2015 values without the added sugar component were 44.25 (0.38) for consumers and 43.03 (0.29) for non-consumers (*p* < 0.0066). For 1999–2018 NHANES, HEI-2015 values were 44.30 (0.21) for consumers and 43.57 (0.16) for nonconsumers (*p* < 0.0013).

Table 4 compares LCS consumers and non-consumers on daily dietary energy, percent of energy from total and added sugar, fiber, and alcohol. Mean daily energy intakes were lower for LCS consumers than for LCS nonconsumers, consistent with observation that more LCS consumers were women. Among women aged >19 years, energy intakes of LCS consumers were also lower compared to LCS non consumers. For the 1999–2018 data, energy intakes for women aged >19 years were 1762 (12) for LCS consumers and 1817 (8) for nonconsumers. Importantly, LCS consumers had significantly lower intakes of added sugars than did nonconsumers. For the 1999–2018 data, the values for all participants were 13.65 tsp eq of added sugars for LCS consumers and 20.7 tsp eq for LCS nonconsumers. For women aged >19 years, the values were 12.10 (0.2) tsp eq for LCS consumers and 17.46 (0.2) tsp eq for LCS nonconsumers. LCS consumers had about 6 fewer tsp eq of added sugars in their diets as compared to nonconsumers. Fiber intakes were higher for LCS consumers and alcohol intake was lower as compared to LCS nonconsumers.

### 3.4. Cancer Mortality Hazard Ratio Associations with Aspartame, Saccharin, and LCS

The NHANES 1988–1994 provides separate intake estimates for aspartame and saccharin. Shown in Table 5 are hazard ratios and upper and lower 95th percentile confidence intervals) for tertiles of each LCS intake by gender and age group (19+, 19–50 and 50+). Nonconsumers were the reference group (HR = 1.0) and the hazard ratio associations were adjusted for age, sex, race/ethnicity, education, current smoking status (Y/N), alcohol consumption, physical activity level, and BMI. For aspartame, higher aspartame intake among consumers was not associated with an increased risk of cancer mortality as compared to nonconsumers. In general, confidence limits for all of the hazard ratio point estimates for any tertile of aspartame intake for any age groups included 1.0 meaning that there were no differences in cancer mortality risk between consumers and nonconsumers within any of the subgroups. There were four hazard ratio point estimates that indicated reduced cancer risk (19+ and 19–50 years gender combined for tertile 1 and 19+ and 51+ years males for tertile 2).

Higher saccharin intake among consumers was not associated with an increased risk of cancer mortality as compared to nonconsumers. In general, confidence limits for all of the hazard ratio point estimates for any tertile of intake for any age groups included 1.0 meaning that there were no differences in cancer mortality risk between consumers and nonconsumers within any of the subgroups. However, in 19–50-years-old males there was an indication that increased saccharin intake was associated with a lower cancer mortality risk.

In combined NHANES data for the years 1988–1994 (Table 6) or 1999–2018 (Table 7), higher LCS intake among consumers was not associated with an increased risk of cancer mortality as compared to nonconsumers. The results for 1988–1994 showed that confidence limits for all of the hazard ratio point estimates for any tertile of LCS for any age groups included 1.0, meaning that there were no differences in cancer mortality risk between consumers and nonconsumers within any of the subgroups. Results for 1988–2018 NHANES indicated there were six hazard ratio point estimates that indicated lower cancer risk (19+ years males for tertile 1 and gender combined for tertile 2, 19–50 years gender combine and males for tertile 1 and females for tertile 2, and 51+ years males for tertile 2. Additionally, higher LCS intake among three groups was associated with a lower risk of cancer mortality (19+ years all, 51+ years all and males).

In all analyses, cancer mortality was consistently and positively associated with age and inversely associated with moderate/vigorous physical activity. Importantly, cancer mortality was consistently and positively associated with current smoking status (Supplemental Tables S1–S3). 

## 4. Discussion

Results from this large and nationally representative study of the US population, based on NHANES data from 1988–1994 and 1999–2018 [25,27], show no association between higher intake of low-calorie sweeteners and overall cancer mortality risk. Data on cancer mortality came from the US 2019 public use linked mortality files [26]. The NHANES 1988–1994 dietary intakes [23] did distinguish between different types of sweetness, aspartame, and saccharin, but the later NHANES cycles did not [27].

The present analyses were conducted separately for men and women and for three different age groups. Results for 1988–1994 and for 1988–2018 showed that confidence limits for all of the hazard ratio point estimates for any tertile of LCS for any age groups included 1.0. In other words, there were no differences in cancer mortality risk between consumers and nonconsumers within any of the subgroups. If anything, data analyses were suggestive of a slight reduction in cancer mortality risk among LCS users within selected subgroups. As might be expected, and serving as a test of our ability to assess mortality differences, cancer mortality was consistently and positively associated with age and inversely associated with moderate or vigorous physical activity. It is important to also note that cancer mortality was consistently and positively associated with current smoking status.

Our analyses confirm previous findings (some also based on NHANES data) that LCS use was associated with higher BMI values and with higher prevalent obesity and type 2 diabetes [3,16]. The NHANES protocols include a medical visit. Both height and weight are measured, along with selected biomarkers, glucose, insulin, and plasma lipids [25], whereas diabetes history is obtained by self-report. In past studies, we used retroactive 10-y weight history—another component of NHANES—to show that previously expressed desire to lose/control body weight was linked to higher LCS use [16]. That study counters the still-repeated arguments that LCS leads to weight gain and is a causal factor in the development of obesity [8,11,12]; assertions that were not confirmed in recent systematic reviews [34,35] It is important to note here that obesity is a recognized risk factor for many cancers that may be unrelated to LCS use.

Our analyses confirm all previous observations linking LCS use to higher socioeconomic status, better health behaviors and higher quality diets [3,16,31]. First, LCS consumers were about 29% of the US population. LCS consumers were more likely to be female, older, with higher education and incomes and from White non-Hispanic groups [16,31]. In some past studies, LCS consumers were more likely to engage in physical activity [31]; that trend was no longer apparent in the more recent data. On the other hand, LCS consumers were significantly less likely to be current smokers [16].

Analyses of HEI-2015 diet quality scores showed that LCS consumers had diets that were more consistent with the Dietary Guidelines for Americans as compared to LCS nonconsumers [33]. The most significant effect was for the added sugars subscore; LCS consumers had added sugar intakes that were significantly lower than those of nonconsumers. Diets of LCS consumers were lower in alcohol, higher in fiber, and higher in a number of desirable food groups, including vegetables, whole fruit, dairy, and whole grains as compared to the diets of LCS nonconsumers. On the other hand, consistent with past observations, diets of LCS consumers were also higher in saturated fat and sodium compared to nonconsumers.

In summary, the present data confirm the previously observed links between LCS use and higher socioeconomic status, less smoking, and better diets [16,31]. As expected, there was an association between LCS use and prevalent obesity and diabetes, always seen in cross-sectional studies. The present data show no association between aspartame, saccharin (1988–1994) and total LCS use (1988–2018) tertiles and overall cancer mortality. This study used regression analyses to compare LCS consumers with nonconsumers and hazard ratio analyses to assess associations with cancer mortality. The latter analyses were adjusted for age, gender, race/ethnicity, education level, current smoking, alcohol intake, physical activity, and measured BMI.

The present results stand in contrast to a recent report based on the French Nutrinet Santé volunteer cohort, a large and predominantly female sample of convenience [23]. As noted by the authors, the NutriNet sample was largely female, of higher educational and professional status, and more likely to engage in health-conscious diet and lifestyle behaviors than the general public [23] It is worth noting that LCS were used by 36.9% of the sample (compared to 29% in the US) and that the mean self-reported BMI was only 23.69, as opposed to 28.02 in the US [23]. Importantly, high LCS users in the Nutrinet Study were more likely to smoke, in stark contrast to data from the US [23]. Furthermore, high LCS users in the Nutrinet study reported higher intakes of regular sugar-sweetened beverages compared to nonusers [23]. Aspartame intake in the Nutrinet study were lower than those seen in the French population [23]. It is clear that LCS consumers in the US and in France had some unique characteristics in terms of both sociodemographic indicators and health behaviors. What is concerning is that those indicators and behaviors appeared to be diametrically opposed.

Our study had strengths and limitations. One strength was the use of a large and nationally representative sample of the US population that was linked to mortality data. In this way, we were able to convert cross-sectional NHANES data into a longitudinal study. We also used numerous covariates to adjust mortality estimates. Even though our analyses adjusted for multiple dietary and lifestyle variables, linear regression modeling has its limitations, and some residual confounding is expected. Our analyses were consistent with other reports on age and lifestyle behaviors and cancer risk [36]. The present data pointed to a protective effect of physical activity and adverse impacts of age and current smoking.

There were limitations. First, the What We Eat In America dietary intakes component of NHANES is based on one or two 24-h dietary recalls. Assigning participants to LCS consumers and nonconsumer groups can be problematic—even two-day intakes are not representative of habitual consumption. In general, randomized controlled trials make for a higher standard of evidence [35,37]. Secondly, LCS beverages and foods are not flagged and need to be searched for in FNDDS, the USDA nutrient composition data file, using custom designed coding. Thirdly, the current FNDDS does not distinguish among different categories of LCS. Only the 1988–1994 data allowed us to distinguish aspartame and saccharin. Finally, the mortality files provided overall cancer mortality only. We were not able to look at specific cancers, such as breast cancer or obesity-related cancers. Finally, all observational studies on diets and health outcomes (or mortality data) may be confounded by any number of unobserved variables, many of which are related to socioeconomic status and lifestyle behaviors.

## 5. Conclusions

The present analyses of nationally representative databases for the US showed the expected links between LCS consumption and higher education and incomes, less smoking, and higher-quality diets. Analyses also showed noncausal cross-sectional association between LCS use and prevalence of obesity and type 2 diabetes. Analyses of linked mortality files failed to show any association between any LCS use, aspartame use or saccharin use and cancer mortality. LCS use was analyzed as total LCS for 1988–2018 and separately for aspartame and saccharin given intakes available in the 1988–1994 data.

## Figures and Tables

**Table 1:** Hazard ratio analyses exclusions table.

Exclusion Description	NHANES III 1988–1994		All 1988–2018	
	Exclusions *n*	Total Exclusions	Exclusions *n*	Total Exclusions
Total sample	19,215		76,324	
Dietary recall incomplete	2882	2882	9418	9418
Pregnant or lactating female	385	3267	2198	11,616
Dietary intake missing kcal = 0	0	3267	6	11,622
Not mortality eligible	10	3277	92	11,714
Told by doctor had diabetes	1292	4569	8145	19,859
Told by doctor had MI *	583	5152	1941	21,800
Told by doctor had CHF *	203	5355	723	22,523
Told by doctor had stroke	230	5585	1118	23,641
Told by doctor had cancer	867	6452	3790	27,431
Education level missing	81	6533	119	27,550
Current smoking missing	1	6534	58	27,608
Physical activity level missing	220	6754	228	27,836
BMI missing	23	6777	424	28,260
Mortality Analysis *n*	12,438		48,064	

* MI, myocardial infarction; CHF, chronic heart failure.

**Table 2:** Participant characteristics for adults >19 years in NHANES 1988–2018. Data are percentages (standard error—SE) and means (SE). Data adjusted for complex sample design of NHANES and used relevant sample weights.

	Characteristics	1988–1994 NHANES	1999–2018 NHANES	1988–2018 NHANES
		***n* = 15,948**	***n* = 48,754**	***n* = 64,702**
	LCS consumers	29.87 (0.54)	29.11 (0.44)	29.26 (0.37)
Gender	Female	51.56 (0.44)	50.76 (0.28)	50.92 (0.24)
Race/ethnicity	NH White	76.36 (1.25)	68.40 (1.03) *	69.92 (0.86)
	NH Black	10.95 (0.63)	11.28 (0.58)	11.22 (0.48)
	MexAmerican	4.98 (0.40)	8.17 (0.54) *	7.56 (0.44)
	Other Hispanic	4.24 (0.63)	5.38 (0.42)	5.16 (0.36)
	Other	3.47 (0.42)	6.77 (0.31) *	6.14 (0.26)
PIR	<1.35	19.22 (0.97)	23.10 (0.58) *	22.36 (0.51)
	1.35–1.85	10.81 (0.52)	9.91 (0.25)	10.08 (0.22)
	>1.85	69.96 (1.12)	66.99 (0.69) *	67.56 (0.60)
Education	<HS	58.98 (1.20)	41.75 (0.68) *	45.04 (0.60)
	Some college	20.73 (0.68)	31.36 (0.39) *	29.33 (0.33)
	>BA	20.29 (0.86)	26.89 (0.70) *	25.63 (0.59)
Physical activity	Sedentary	21.55 (0.74)	26.88 (0.44) *	25.88 (0.38)
	Moderate	44.41 (0.71)	33.86 (0.38) *	35.85 (0.33)
	Vigorous	34.04 (0.78)	39.25 (0.52) *	38.27 (0.44)
Smoking, current		28.48 (0.82)	20.67 (0.45) *	22.17 (0.39)
Obesity		22.06 (0.71)	35.35 (0.45) *	32.78 (0.40)
Diabetes		5.36 (0.25)	10.71 (0.20) *	9.68 (0.18)
		Mean (SE)	Mean (SE)	Mean (SE)
Age (y)		44.52 (0.48)	47.01 (0.20) *	46.53 (0.18)
BMI (kg/m^2^)		26.50 (0.11)	28.72 (0.07) *	28.29 (0.06)

* Statistically different from NHANES 1988–1994 to 1999–2018, *p* < 0.05.

**Table 3:** Low calorie sweetener (LCS) consumers and nonconsumers by demographics and health outcomes. Data are percentages (standard error, SE) and means (SE). Data adjusted for complex sample design of NHANES using relevant sample weights.

	Variables	NHANES 1988–1994			NHANES 1999–2018		
		Consumers	Nonconsumers		Consumers	Nonconsumers	
		***n* = 3979 (29.87%)**	***n* = 11,969 (70.13%)**		***n* = 12,474 (29.11%)**	***n* = 36,280 (70.89%)**	
Gender	Female	60.21 (1.09)	47.87 (0.56)	<0.0001	57.08 (0.58)	48.17 (0.35)	<0.0001
Race/ethnicity	White-NH	86.53 (0.73)	72.03 (1.52)	<0.0001	79.91 (0.86)	63.67 (1.13)	<0.0001
	Black-NH	6.87 (0.53)	12.69 (0.72)	<0.0001	6.62 (0.40)	13.19 (0.67)	<0.0001
	Mex. American	2.85 (0.20)	5.89 (0.54)	<0.0001	5.34 (0.45)	9.33 (0.60)	<0.0001
	Other Hispanic	2.74 (0.44)	4.88 (0.79)	0.0041	3.66 (0.33)	6.09 (0.48)	<0.0001
PIR	<1.35	11.86 (0.84)	22.39 (1.16)	<0.0001	14.80 (0.57)	26.57 (0.66)	<0.0001
	1.35–1.85	8.46 (0.69)	11.82 (0.59)	<0.0001	7.79 (0.33)	10.79 (0.29)	<0.0001
	>1.85	79.67 (1.23)	65.79 (1.30)	<0.0001	77.41 (0.72)	62.64 (0.77)	<0.0001
Education	<HS	49.89 (1.86)	62.87 (1.28)	<0.0001	35.08 (0.87)	44.49 (0.72)	<0.0001
	Some college	23.21 (1.26)	19.67 (0.80)	0.0227	31.82 (0.63)	31.17 (0.48)	0.4098
	BA	26.90 (1.43)	17.46 (0.95)	<0.0001	33.11 (1.00)	24.34 (0.70)	<0.0001
Physical activity	Sedentary	21.88 (1.06)	21.42 (0.80)	0.6729	26.22 (0.62)	27.15 (0.49)	0.1530
	Moderate	43.64 (0.90)	44.74 (0.84)	0.3147	36.78 (0.59)	32.67 (0.47)	<0.0001
	Vigorous	34.48 (1.25)	33.85 (0.81)	0.6075	37.00 (0.74)	40.18 (0.58)	0.0001
Smoking	Current smokers	20.42 (1.08)	31.91 (0.89)	<0.0001	15.40 (0.48)	22.84 (0.54)	<0.0001
Obesity	Prevalence	29.34 (1.31)	18.96 (0.65)	<0.0001	43.14 (0.75)	32.15 (0.50)	<0.0001
Diabetes	Prevalence	11.92 (0.82)	2.57 (0.20)	<0.0001	19.18 (0.48)	7.23 (0.18)	<0.0001
BMI	Kg/m^2^	27.64 (0.16)	26.01 (0.11)	<0.0001	30.03 (0.10)	28.18 (0.07)	<0.0001
Age	Mean age years	46.96 (0.80)	43.48 (0.48)	<0.0001	51.50 (0.26)	45.16 (0.20)	<0.0001
LCS food, beverage	Mean LCS grams/day	469 (13)	0	<0.0001	568 (9)	0	<0.0001

**Table 4:** LCS consumers and non-consumers by dietary and health variables. Data are means (standard error, SE). Data adjusted for complex sample design of NHANES and used relevant sample weights.

Variables	NHANES 1988–1994			NHANES 1999–2018		
	Consumers	Nonconsumers		Consumers	Nonconsumers	
***n***	***n* = 3979**	***n* = 11,969**		***n* = 12,474**	***n* = 36,280**	
Energy. kcal/day	2037 (29)	2259 (21)	<0.0001	2045 (12)	2213 (9)	<0.0001
HEI 2015	51.88 (0.41)	49.09 (0.33)	<0.0001	51.91 (0.23)	49.55 (0.19)	<0.0001
Added sugar tsp eq/day	13.71 (0.39)	20.20 (0.43)	<0.0001	13.65 (0.18)	20.70 (0.20)	<0.0001
Alcohol g/day	8.11 (0.59)	11.81 (0.70)	<0.0001	8.92 (0.38)	11.62 (0.30)	<0.0001
Fiber g/day	17.11 (0.30)	16.54 (0.15)	0.0952	16.95 (0.16)	16.39 (0.13)	0.0014

**Table 5:** Cancer mortality hazard ratio (HR) associations with aspartame and saccharin consumption tertiles, 1988–1994. Data are hazard ratios (HR) and 95th percentile confidence lower (LCL) and upper (UCL) levels. Cox proportional hazards regression was used to estimate HR and 95th percentile confidence levels for cancer mortality. HRs were estimated by assigning nonconsumers of aspartame/saccharin/LCS to the reference group (HR = 1.0). LCS consumers were then split by tertiles of consumption T1, T2, and T3, using SAS 9.4 PROC SURVEYPHREG with age, sex, race/ethnicity, education, current smoking status (Y/N), alcohol consumption, physical activity level, and BMI (continuous) as covariates. Data adjusted for complex sample design of NHANES and used relevant sample weights.

LCS Type	Age	Gender	*n*	Event *n*	Tertial 1	Tertile 2	Tertile 3	Group Trend	
					HR (LCL, UCL)	HR (LCL, UCL)	HR (LCL, UCL)	Beta (LCL, UCL)	*p*
Aspartame (mg)	19–50	All	8511	322	0.38 (0.18, 0.82)	0.57 (0.27, 1.23)	1.54 (0.91, 2.62)	1.03 (0.83, 1.27)	0.7825
		Male	4133	162	0.44 (0.17, 1.14)	1.19 (0.51, 2.77)	1.46 (0.55, 3.89)	1.10 (0.81, 1.50)	0.5494
		Female	4378	160	0.35 (0.11, 1.16)	0.46 (0.18, 1.17)	1.28 (0.65, 2.50)	0.95 (0.74, 1.23)	0.7151
	51+	All	3927	656	0.86 (0.61, 1.23)	0.65 (0.37, 1.14)	0.99 (0.68, 1.44)	0.95 (0.85, 1.08)	0.4396
		Male	1862	384	0.72 (0.38, 1.36)	0.28 (0.13, 0.56)	1.01 (0.56, 1.81)	0.84 (0.68, 1.03)	0.0884
		Female	2065	272	1.06 (0.69, 1.63)	0.95 (0.48, 1.88)	1.24 (0.78, 1.97)	1.06 (0.91, 1.22)	0.4677
	19+	All	12,438	978	0.70 (0.49, 0.99)	0.72 (0.49, 1.06)	1.32 (0.94, 1.85)	1.00 (0.89, 1.13)	0.9755
		Male	5995	546	0.61 (0.37, 1.02)	0.55 (0.32, 0.94)	1.50 (0.74, 3.05)	0.98 (0.77, 1.24)	0.8547
		Female	6443	432	0.83 (0.52, 1.34)	0.81 (0.52, 1.26)	1.15 (0.81, 1.65)	1.00 (0.89, 1.12)	0.9777
Saccharin (mg)	19–50	All	8511	322	1.09 (0.51, 2.33)	1.32 (0.68, 2.56)	0.91 (0.50, 1.65)	1.03 (0.87, 1.23)	0.7394
		Male	4133	162	0.69 (0.20, 2.39)	2.29 (1.12, 4.66)	0.50 (0.13, 1.85)	1.07 (0.82, 1.40	0.6116
		Female	4378	160	1.48 (0.60, 3.68)	0.75 (0.36, 1.54)	1.03 (0.45, 2.37)	0.98 (0.78, 1.24)	0.8741
	51+	All	3927	656	0.90 (0.57, 1.43)	0.87 (0.59, 1.29)	0.77 (0.47, 1.25)	0.92 (0.81, 1.05	0.2201
		Male	1862	384	1.26 (0.80, 1.97)	0.55 (0.24, 1.25)	0.51 (0.24, 1.05)	0.81 (0.69, 0.96)	0.0150
		Female	2065	272	0.73 (0.39, 1.36)	0.90 (0.48, 1.71)	1.20 (0.75, 1.91)	1.02 (0.86, 1.20)	0.8433
	19+	All	12,438	978	1.11 (0.77, 1.59)	0.96 (0.61, 1.51)	0.84 (0.57, 1.25)	0.96 (0.86, 1.08)	0.5084
		Male	5995	546	1.03 (0.71, 1.50)	1.14 (0.57, 2.28)	0.63 (0.33, 1.20)	0.93 (0.79, 1.10)	0.4115
		Female	6443	432	1.07 (0.60, 1.90)	0.90 (0.57, 1.43)	0.98 (0.63, 1.53)	0.99 (0.86, 1.12)	0.8180

**Table 6:** Cancer mortality hazard ratio associations with low-calorie sweeteners, 1988–1994. Data are hazard ratios (HR) and 95th percentile confidence lower (LCL) and upper (UCL) levels. Cox proportional hazards regression was used to estimate HR and 95th percentile confidence levels for cancer mortality. HRs were estimated by assigning nonconsumers of aspartame/saccharin/LCS to the reference group (HR = 1.0). LCS consumers were then split by tertiles of consumption T1, T2, and T3, using SAS 9.4 PROC SURVEYPHREG with age, sex, race/ethnicity, education, current smoking status (Y/N), alcohol consumption, physical activity level, and BMI (continuous) as covariates. Data adjusted for complex sample design of NHANES and used relevant sample weights.

Age	Gender	*n*	Event *n*	Tertile 1	Tertile 2	Tertile 3	Group Trend	
				HR (LCL, UCL)	HR (LCL, UCL)	HR (LCL, UCL)	Beta (LCL, UCL)	*p*
19–50	All	8511	322	0.75 (0.37, 1.49)	0.83 (0.44, 1.56)	1.29 (0.77, 2.16)	1.03 (0.87, 1.22)	0.7328
	Male	4133	162	0.34 (0.11, 1.12)	1.46 (0.69, 3.08)	0.86 (0.25, 3.02)	1.01 (0.75, 1.37)	0.9330
	Female	4378	160	1.32 (0.61, 2.85)	0.51 (0.24, 1.10)	1.26 (0.62, 2.54)	0.98 (0.79, 1.23)	0.8746
51+	All	3927	656	0.95 (0.68, 1.31)	0.80 (0.58, 1.08)	0.95 (0.19, 1.50)	0.94 (0.84, 1.06)	0.3349
	Male	1862	384	0.86 (0.57, 1.31)	0.64 (0.36, 1.16)	0.78 (0.40, 1.53)	0.87 (0.73, 1.03)	0.0926
	Female	2065	272	1.02 (0.59, 1.75)	0.93 (0.63, 1.38)	1.20 (0.70, 2.05)	1.02 (0.88, 1.18)	0.8492
19+	All	12,438	978	0.99 (0.72, 1.36)	0.73 (0.52, 1.03)	1.16 (0.85, 1.57)	0.98 (0.89, 1.09)	0.6871
	Male	5995	546	0.67 (0.45, 1.00)	0.86 (0.50, 1.49)	1.07 (0.54, 2.11)	0.96 (0.79, 1.16)	0.6555
	Female	6443	432	1.17 (0.68, 1.98)	0.80 (0.56, 1.14)	1.16 (0.77, 1.75)	1.00 (0.89, 1.13)	0.9817

**Table 7:** Cancer mortality hazard ratio associations with low-calorie sweeteners, 1988–2018. Data are hazard ratios (HR) and 95th percentile confidence lower (LCL) and upper (UCL) levels. Cox proportional hazards regression was used to estimate HR and 95th percentile confidence levels for cancer mortality. HRs were estimated by assigning nonconsumers of aspartame/saccharin/LCS to the reference group (HR = 1.0). LCS consumers were then split by tertiles of consumption T1, T2, and T3, using SAS 9.4 PROC SURVEYPHREG with age, sex, race/ethnicity, education, current smoking status (Y/N), alcohol consumption, physical activity level, and BMI (continuous) as covariates. Data adjusted for complex sample design of NHANES and used relevant sample weights.

Age	Gender	*n*	Event *n*	Tertile 1	Tertile 2	Tertile 3	Group Trend	
				HR (LCL, UCL)	HR (LCL, UCL)	HR (LCL, UCL)	Beta (LCL, UCL)	*p*
19–50	All	31,565	458	0.61 (0.33, 1.13)	0.68 (0.40, 1.16)	0.89 (0.53, 1.51)	0.91 (0.77, 1.08)	0.2791
	Male	16,057	232	0.29 (0.10, 0.84)	1.22 (0.65, 2.28)	0.63 (0.21, 1.89)	0.92 (0.71, 1.19)	0.5133
	Female	15,508	226	0.99 (0.50, 1.97)	0.38 (0.19, 0.78)	0.86 (0.42, 1.80)	0.86 (0.68, 1.10)	0.2309
51+	All	16,499	1332	0.84 (0.67, 1.06)	0.84 (0.67, 1.06)	0.77 (0.57, 1.05)	0.91 (0.84, 1.00)	0.0446
	Male	7753	793	0.81 (0.57, 1.14)	0.65 (0.45, 0.93)	0.74 (0.46, 1.21)	0.87 (0.76, 0.99)	0.0352
	Female	8746	539	0.89 (0.65, 1.22)	0.99 (0.71, 1.37)	0.95 (0.61, 1.47)	0.98 (0.53, 1.51)	0.7680
19+	All	48,064	1790	0.81 (0.65, 1.01)	0.79 (0.63, 0.99)	0.85 (0.64, 1.11)	0.92 (0.88, 1.10)	0.0382
	Male	23,810	1025	0.68 (0.50, 0.91)	0.75 (0.53, 1.07)	0.85 (0.54, 1.34)	0.90 (0.79 1.02)	0.1010
	Female	24,254	765	0.91 (0.64, 1.29)	0.87 (0.64, 1.18)	0.82 (0.54, 1.23)	0.93 (0.83, 1.05)	0.2436

## Data Availability

All NHANES data are publicly available on the NCHS and USDA websites. All documentation is provided online at https://www.cdc.gov/nchs/nhanes/index.htm (accessed on 20 November 2022).

## Supplementary Materials

The following supporting information can be downloaded at: https://www.mdpi.com/article/10.3390/nu14234957/s1, Table S1: Cancer Mortality Beta Coefficients for Associations with Aspartame and Saccharin, 1988–1994; Table S2: Cancer Mortality Beta Coefficients for Associations with Low Calorie Sweeteners, 1988–1994; Table S3: Cancer Mortality Beta Coefficients for Associations with Low Calorie Sweeteners, 1988–2018.
