# Supplementary material for: No Association between Low-Calorie Sweetener (LCS) Use and Overall Cancer Risk in the Nationally Representative Database in the US: Analyses of NHANES 1988–2018 Data and 2019 Public-Use Linked Mortality Files
Source: Nutrients. 2022 Nov 22;14(23):4957. doi: 10.3390/nu14234957 (PMC9740385; doi:10.3390/nu14234957)
Supplement: Supplementary file 1 [file nutrients-14-04957-s001.zip › nutrients-2016674-supplementary.pdf]

SUPPLEMENTAL TABLES S1-S3

No association between low-calorie sweetener (LCS) use and overall cancer risk in the nationally representative database in the US: Analyses of NHANES 1988-2018 data and 2019 Pub-lic-Use Linked Mortality Files

Victor L. Fulgoni III[1] and Adam Drewnowski [2]*

[1] Nutrition Impact LLC, Battle Creek, MI 49014, USA; vic3rd@aol.com
[3] Center for Public Health Nutrition, University of Washington, Box 353410, Seattle, WA 98195, USA
* Correspondence: adamdrew@uw.edu

Supplemental Table S1. Cancer Mortality Beta Coefficients for Associations with Aspartame and Saccharin, 1988-1994.

		19+ y		19-50 y		51+ y	
**Modeling Variable**	**levels**	**HR (LCL 95, UCL 95)**	**P**	**HR (LCL 95, UCL 95)**	**P**	**HR (LCL 95, UCL 95)**	**P**
ASPARTAME							
Age		1.09 (1.08, 1.10)	**<0.0001**	1.10 (1.09, 1.12)	**<0.0001**	1.07 (1.05, 1.08)	**<0.0001**
Aspartame Intake	Quartile = 1	ref					
	Quartile = 2	0.70 (0.49, 0.99)	0.0448	0.38 (0.18, 0.81)	0.0136	0.86 (0.61, 1.23)	0.4122
	Quartile = 3	0.72 (0.49, 1.06)	0.0951	0.57 (0.26, 1.23)	0.1475	0.65 (0.37, 1.14)	0.1304
	Quartile = 4	1.32 (0.94, 1.85)	0.1055	1.54 (0.90, 2.61)	0.1104	0.99 (0.68, 1.44)	0.9739
Gender	Female	ref		ref		ref	
	Male	1.34 (1.10, 1.63)	**0.0040**	1.03 (0.70, 1.52)	0.8656	1.56 (1.29, 1.89)	**<0.0001**
Ethnicity	NH white	ref					
	Black	1.04 (0.88, 1.23)	0.6300	1.01 (0.78, 1.32)	0.9126	1.09 (0.86, 1.39)	0.4478
	Mexican American	0.74 (0.61, 0.91)	**0.0057**	0.67 (0.45, 0.99)	0.0469	0.83 (0.61, 1.13)	0.2258
	Other Hispanic	0.46 (0.22, 0.96)	0.0383	0.23 (0.06, 0.94)	0.0405	0.63 (0.28, 1.40)	0.2534
	Other	0.68 (0.33, 1.37)	0.2734	0.67 (0.20, 2.24)	0.5076	0.71 (0.30, 1.71)	0.4403
Education	≤ HS	ref					
	Some College	1.11 (0.86, 1.44)	0.4181	0.67 (0.42, 1.06)	0.0876	1.59 (1.19, 2.12)	**0.0022**
	≥ Bachelors Degree	0.80 (0.62, 1.03)	0.0800	0.75 (0.50, 1.14)	0.1781	0.80 (0.56, 1.13)	0.1967
Current Smoking	No	ref		REF		ref	
	Yes	2.52 (2.03, 3.13)	**<0.0001**	2.21 (1.57, 3.11)	**<0.0001**	2.55 (1.98, 3.27)	**<0.0001**
Alcohol (iui gm)	continuous	1.00 (1.00, 1.01)	0.0886	1.00 (1.00, 1.01)	0.0979	1.00 (0.99, 1.01)	0.8245
Physical Activity	Sedentary	ref					
	Moderate	0.79 (0.64, 0.99)	0.0402	0.71 (0.51, 0.99)	0.0438	0.89 (0.67, 1.20)	0.4443
	Vigorous	0.58 (0.44, 0.75)	**0.0001**	0.56 (0.35, 0.89)	0.0157	0.64 (0.48, 0.85)	**0.0027**
BMI (kg/m**2)	Continuous	1.02 (1.00, 1.04)	0.0444	1.02 (0.99, 1.05)	0.2639	1.01 (0.99, 1.04)	0.2518

SACCHARIN							
	Age	1.09 (1.08, 1.09)	**<0.0001**	1.10 (1.08, 1.12)	**<0.0001**	1.06 (1.05, 1.08)	**<0.0001**
Gender	Female	REF					
	Male	1.35 (1.11, 1.64)	**0.0030**	1.10 (0.75, 1.60)	0.6239	1.55 (1.28, 1.88)	**<0.0001**
Saccharin Intake	Quartile = 1	REF					
	Quartile = 2	1.11 (0.77, 1.59)	0.5773	1.09 (0.51, 2.32)	0.8205	0.90 (0.57, 1.43)	0.6574
	Quartile = 3	0.96 (0.61, 1.51)	0.8458	1.32 (0.68, 2.56)	0.4028	0.87 (0.59, 1.29)	0.4855
	Quartile = 4	0.84 (0.57, 1.24)	0.3836	0.91 (0.50, 1.65)	0.7524	0.77 (0.47, 1.25)	0.2797
Ethnicity	White	REF					
	Black	1.04 (0.88, 1.24)	0.6275	1.03 (0.78, 1.36)	0.8287	1.10 (0.87, 1.41)	0.4210
	Mexican American	0.75 (0.61, 0.92)	**0.0062**	0.69 (0.46, 1.04)	0.0730	0.83 (0.61, 1.13)	0.2294
	Other Hispanic	0.46 (0.22, 0.97)	0.0413	0.25 (0.06, 0.96)	0.0433	0.63 (0.28, 1.40)	0.2489
	Other	0.69 (0.34, 1.41)	0.3042	0.72 (0.21, 2.43)	0.5911	0.71 (0.30, 1.72)	0.4426
Education	≤ HS	REF					
	Some College	1.11 (0.85, 1.44)	0.4419	0.67 (0.42, 1.06)	0.0835	1.59 (1.19, 2.11)	**0.0022**
	≥ Bachelors Degree	0.79 (0.62, 1.02)	0.0689	0.70 (0.48, 1.04)	0.0759	0.80 (0.57, 1.12)	0.1917
Current Smoking	No	REF					
	Yes	2.56 (2.07, 3.17)	**<0.0001**	2.27 (1.63, 3.16)	**<0.0001**	2.58 (2.01, 3.30)	**<0.0001**
Alcohol (iui gm)	continuous	1.00 (1.00, 1.01)	0.0955	1.00 (1.00, 1.01)	0.0994	1.00 (0.99, 1.01)	0.7330
Physical Activity	Sedentary	REF					
	Moderate	0.80 (0.64, 1.00)	0.0461	0.71 (0.51, 0.99)	0.0426	0.90 (0.67, 1.22)	0.4926
	Vigorous	0.58 (0.44, 0.75)	**0.0001**	0.55 (0.35, 0.87)	0.0121	0.64 (0.48, 0.86)	**0.0035**
BMI (kg/m**2)	Continuous	1.02 (1.00, 1.04)	0.0490	1.02 (0.98, 1.05)	0.3039	1.01 (0.99, 1.04)	0.2401

iui: individual usual intake

Supplemental Table S2. Cancer Mortality Beta Coefficients for Associations with Low Calorie Sweeteners, 1988-1994.

		**19+ y**		**19-50 y**		**51+ y**	
	**Modeling Variable**	**HR (LCL 95, UCL 95)**	**P**	**HR (LCL 95, UCL 95)**	**P**	**HR (LCL 95, UCL 95)**	**P**
	Age	1.09 (1.08, 1.09)	**<0.0001**	1.10 (1.08, 1.12)	**<0.0001**	1.07 (1.05, 1.08)	**<0.0001**
LCS Intake	Quartile = 1	**REF**					
	Quartile = 2	0.96 (0.70, 1.31)	0.8044	0.75 (0.38, 1.48)	0.4039	0.90 (0.65, 1.24)	0.5022
	Quartile = 3	0.75 (0.54, 1.05)	0.0963	0.82 (0.43, 1.58)	0.5524	0.78 (0.56, 1.08)	0.1308
	Quartile = 4	1.15 (0.85, 1.57)	0.3600	1.29 (0.77, 2.15)	0.3308	1.04 (0.69, 1.56)	0.8568
Gender	Female	REF					
	Male	1.34 (1.10, 1.62)	**0.0042**	1.06 (0.73, 1.54)	0.7419	1.56 (1.28, 1.89)	**<0.0001**
Ethnicity	White	REF					
	Black	1.04 (0.88, 1.24)	0.6350	1.02 (0.77, 1.34)	0.8883	1.10 (0.86, 1.40)	0.4502
	Mexican American	0.75 (0.61, 0.91)	**0.0058**	0.68 (0.46, 1.02)	0.0590	0.83 (0.61, 1.13)	0.2345
	Other Hispanic	0.46 (0.22, 0.97)	0.0408	0.24 (0.06, 0.95)	0.0428	0.63 (0.28, 1.41)	0.2548
	Other	0.68 (0.33, 1.39)	0.2869	0.69 (0.21, 2.27)	0.5303	0.71 (0.30, 1.72)	0.4435
Education	≤ HS	REF					
	Some College	1.10 (0.85, 1.43)	0.4568	0.67 (0.42, 1.07)	0.0928	1.58 (1.19, 2.11)	**0.0023**
	≥ Bachelors Degree	0.80 (0.62, 1.02)	0.0742	0.74 (0.49, 1.10)	0.1344	0.79 (0.56, 1.12)	0.1745
Current Smoking	No	REF					
	Yes	2.54 (2.06, 3.14)	**<0.0001**	2.25 (1.61, 3.13)	**<0.0001**	2.57 (2.00, 3.30)	**<0.0001**
	Alcohol (iui gm)	1.00 (1.00, 1.01)	0.1052	1.00 (1.00, 1.01)	0.0983	1.00 (0.99, 1.01)	0.8066
Physical Activity Level	Sedentary	REF					
	Moderate	0.80 (0.64, 1.00)	0.0500	0.71 (0.51, 0.99)	0.0434	0.91 (0.68, 1.23)	0.5292
	Vigorous	0.58 (0.45, 0.76)	**0.0001**	0.56 (0.35, 0.89)	0.0156	0.64 (0.49, 0.86)	**0.0031**
	BMI (kg/m**2)	1.02 (1.00, 1.04)	0.0402	1.02 (0.99, 1.05)	0.2833	1.01 (0.99, 1.04)	0.2427

iui: individual usual intakeSupplemental

Supplemental Table S3. Cancer Mortality Beta Coefficients for Associations with Low Calorie Sweeteners, 1988-2018.

		**19+ y**		**19-50 y**		**51+ y**	
	**Modeling Variable**	**HR (LCL 95, UCL 95)**	**P**	**HR (LCL 95, UCL 95)**	**P**	**HR (LCL 95, UCL 95)**	**P**
	Age	1.09 (1.08, 1.09)	**<0.0001**	1.11 (1.09, 1.13)	**<0.0001**	1.07 (1.06, 1.08)	**<0.0001**
LCS Intake	Quartile = 1						
	Quartile = 2	0.81 (0.65, 1.01)	0.0638	0.61 (0.33, 1.13)	0.1137	0.84 (0.67, 1.06)	0.1378
	Quartile = 3	0.79 (0.63, 0.99)	0.0447	0.68 (0.40, 1.16)	0.1584	0.84 (0.67, 1.06)	0.1463
	Quartile = 4	0.85 (0.64, 1.11)	0.2271	0.89 (0.53, 1.51)	0.6747	0.77 (0.57, 1.05)	0.1032
Gender	Female	REF					
	Male	1.50 (1.30, 1.74)	**<0.0001**	0.97 (0.72, 1.31)	0.8327	1.80 (1.53, 2.11)	**<0.0001**
Ethnicity	White	REF					
	Black	1.14 (0.98, 1.32)	0.0850	1.08 (0.85, 1.38)	0.5096	1.16 (0.96, 1.40)	0.1144
	Mexican American	0.80 (0.65, 0.98)	0.0301	0.66 (0.48, 0.90)	**0.0090**	0.89 (0.68, 1.18)	0.4256
	Other Hispanic	0.63 (0.44, 0.90)	0.0105	0.18 (0.06, 0.53)	**0.0018**	0.90 (0.63, 1.29)	0.5669
	Other	0.76 (0.49, 1.18)	0.2157	0.57 (0.24, 1.39)	0.2166	0.86 (0.51, 1.45)	0.5631
Education	≤ HS	REF					
	Some College	0.94 (0.79, 1.13)	0.5254	0.67 (0.48, 0.96)	0.0278	1.06 (0.86, 1.30)	0.5744
	≥ Bachelors Degree	0.88 (0.73, 1.06)	0.1777	0.61 (0.43, 0.88)	**0.0075**	0.96 (0.76, 1.21)	0.7241
Current smoking	No	Ref					
	Yes	2.68 (2.29, 3.13)	**<0.0001**	2.30 (1.75, 3.00)	**<0.0001**	2.65 (2.19, 3.21)	**<0.0001**
Alcohol (iui gm)		1.00 (1.00, 1.01)	0.1612	1.00 (1.00, 1.01)	0.2266	1.00 (1.00, 1.01)	0.4479
Physical Activity	Sedentary	Ref					
	Moderate	0.88 (0.75, 1.03)	0.1151	0.67 (0.50, 0.89)	**0.0059**	0.99 (0.81, 1.22)	0.9385
	Vigorous	0.74 (0.61, 0.90)	**0.0026**	0.61 (0.42, 0.87)	**0.0068**	0.84 (0.68, 1.04)	0.1093
	BMI (kg/m**2)	1.01 (1.00, 1.02)	0.2281	1.01 (0.98, 1.03)	0.6399	1.00 (0.99, 1.02)	0.5685

iui: individual usual intake
